# Successful pregnancy using oral DHEA treatment for hypoandrogenemia in a 30-year-old female with 5 recurrent miscarriages, including fetal demise at 24 weeks: a case report

**DOI:** 10.3389/fmed.2024.1358563

**Published:** 2024-02-15

**Authors:** Phil C. Boyle, Codruta Pandalache, Craig Turczynski

**Affiliations:** NeoFertility Clinic, Dublin, Ireland

**Keywords:** hypoandrogenemia, DHEA treatment, recurrent miscarriage, pregnancy, case report, restorative reproductive medicine

## Abstract

Hypoandrogenemia is not usually considered as a potential cause of recurrent miscarriage. We present the case of a 30-year-old female with 6 previous pregnancies resulting in one live birth and 5 pregnancy losses, including fetal demise at 24 weeks gestation. She had standard investigations after her 4th loss, at a specialized miscarriage clinic. Lupus anticoagulant, anticardiolipin antibodies, thyroid function, parental karyotypes were all normal. Fetal products confirmed triploidy for her 4th miscarriage at 16 weeks gestation. She was reassured and advised to conceive again but had fetal demise after 24 weeks gestation. This was her 5th pregnancy loss with no explanation. She attended our Restorative Reproductive Medicine (RRM) clinic in January 2022. In addition to poor follicle function, we found hypoandrogenemia for the first time. Treatment included follicle stimulation with clomiphene and DHEA 25 mg twice daily pre-conception with DHEA 20 mg once daily maintained throughout pregnancy. She delivered a healthy baby boy by cesarean section at 36 weeks gestation in November 2023. Hypoandrogenemia should be considered as a contributory factor for women with recurrent miscarriage or late pregnancy loss. Restoration of androgens to normal levels with oral DHEA is safe and can improve pregnancy outcome.

## 1 Introduction

Recurrent pregnancy loss, or recurrent miscarriage, is a devastating experience defined by the Royal College of Obstetricians and Gynecologist and the World Health Organization as 3 or more consecutive first trimester losses (1, 2). The American Society for Reproductive Medicine and the European Society for Human Reproduction and Embryology define recurrent pregnancy loss as spontaneous loss of two or more pregnancies, but not necessarily consecutive (2, 3). Recurrent miscarriage using the definition of 2 or more losses affects at least 2% of all couples (4) and extensive investigations will fail to find an explanation for about 50% of the cases (5). This case identified hypoandrogenemia as a potential contributory factor for repeated miscarriage after typical investigations for recurrent miscarriage were normal.

Hypoandrogenemia, also called female androgen insufficiency syndrome (FAIS) (6), results in reduced libido, diminished wellbeing and lowered mood. The likelihood of this condition increases as the woman ages and enters into menopause but can also occur in premenopausal women as ovarian function declines, resulting in low levels of testosterone and DHEAS (7).

Supplementation with DHEA has been demonstrated to increase spontaneous pregnancy rates (8) and is also suggested to improve success with IVF (9). Considered a weak androgen, DHEA is produced by the adrenal gland and the ovaries in women. It functions as a steroid precursor for conversion to testosterone and estradiol (10). Recently DHEA has also been shown to increase serum estradiol during pregnancy and reduce the incidence of miscarriage (11). During the first 8–10 weeks of pregnancy the primary source of estradiol is the corpus luteum, utilizing maternal sources of DHEA. The fetal adrenal gland then becomes the major source of DHEA around 10 weeks of pregnancy, and it is the primary precursor for estradiol produced by the placenta (12). There is increasing evidence for the need of adequate levels of estradiol during pregnancy and the importance of both maternal and fetal sources of androgen precursors (13).

Clinically our Restorative Reproductive Medicine (14) clinic has treated pre-conception hypoandrogenemia and low estrogen during pregnancy with oral DHEA since 2015. Consistent with that approach, our goals for this case were to detect and treat hypoandrogenemia pre-conception and maintain adequate estradiol during pregnancy with DHEA to reduce the risk of miscarriage and late pregnancy loss.

## 2 Case presentation

### 2.1 Patient information

This patient first attended our RRM clinic in January 2022. She is originally from Romania and needed to communicate through a translator. She was 30 years old, and her husband was 35 years. They were both manual workers and she was working as a cleaner. Both were healthy, non-smokers and neither had any previous sexual partners. They denied alcohol or illicit drug use. She was not taking any medications and never used hormonal contraception.

The relevant family history included a mother who had 3 normal pregnancies with no complications. She had 2 siblings, one single brother and one sister who had 2 normal pregnancies.

**FIGURE 1:**
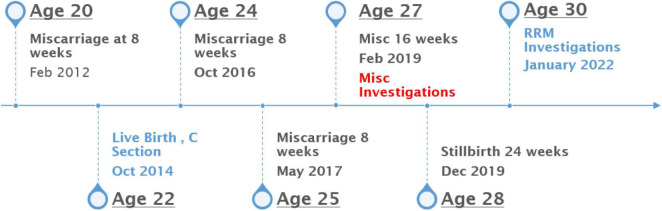
Historical timeline.

She had a history of 6 pregnancies with 1 live birth and 5 miscarriages, including fetal demise at 24 weeks gestation. They were married in 2011 and had their first miscarriage in Feb 2012, at 8 weeks gestation, when she was 20 years old. They spontaneously conceived again, without treatment and had a successful pregnancy delivered by cesarean section at full term in October 2014. This was followed by 4 additional losses as listed in Figure 1.

After the 4th pregnancy loss at 16 weeks gestation in February 2019 she had standard blood tests at a Dublin hospital recurrent miscarriage clinic, as listed in Table 1, including parental karyotyping. No explanation for recurrent miscarriage was found.

**TABLE 1:** Previous investigations from the recurrent miscarriage clinic.

Recurrent miscarriage clinic				
	**Tested**	**Not tested**	**Normal result**	**Abnormal result**
FBC	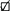		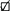	
PT	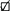			12.6 s (9.6–12.9)
APTT	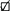		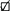	
Lupus screen	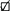		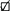	
Thrombophilia screen		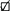		
Prothrombin gene mutation		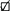		
Vitamin B_12_		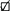		
Folate		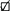		
Anticardiolipin antibodies IgG	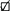		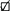	
Anticardiolipin antibodies IgM	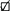		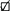	
Beta 2 glycoprotein IgG	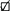		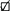	
Beta 2 glycoprotein IgM	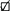		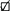	
Free T_4_	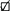		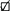	
TSH	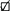		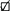	TSH: 2.0 mIU/L
TPO antibodies		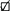		
HSG		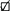		
Hysteroscopy		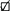		
Patient karyotype	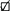		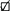	
Partner karyotype	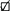		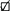	

They were reassured and advised to conceive again with no diagnosis and no treatment recommended. She conceived for the 6th time and had her 5th pregnancy loss, with no fetal heartbeat detectable at 24 weeks gestation. Fetal postmortem examination documented normal anatomy and normal chromosomes.

The couple came to our clinic seeking a reason for their miscarriages and hoped to receive treatment to reduce the risk of another miscarriage.

### 2.2 Clinical findings

She reported a regular 28–30-day cycle, with 4–5 days of bleeding each cycle. Her BMI was 22.8 (57 kg, 1.58 m). She had symptoms of fatigue, low mood, anxiety, and dysmenorrhea—consistent with clinical endorphin deficiency (15).

### 2.3 Diagnostic assessment

She was taught how to track her fertility cycle by a trained NeoFertility Advisor using a specialized fertility app (16). This is essential with RRM treatment to reliably document her cycle and identify her DPO (day of presumed ovulation) to ensure accurate timing of blood tests and ultrasound scans. She had multiple blood tests on day 3 of the menstrual cycle and an additional blood test for progesterone and estradiol day 7 after DPO, to assess “the quality of ovulation.” Results of blood tests are listed in Table 2.

**TABLE 2:** Blood test results.

	Day 3	Day 7 post-ovulation	
**Blood test**	**February 22**	**March 22**	**Normal range**
FSH	7.2		3.5–12.5 miu/ml
LH	6		2.4–12.6 miu/ml
Prolactin	587		25–629 miu/ml
TSH	1.56		0.27–4.20 miu/l
T4	15.9		12.0–22.0 pmol/l
T3	4.9		3.1–6.8 pmol/l
Anti TPO antibodies	<11		0–34 kIU/l
Anti -tTG antibodies	<0.2		0–6.9 U/ml
25 OH Vitamin D	105		30–125 nmol/l
HbA1c	28		29–42 mmol/mol
Hemoglobin	14.7		11.3–15.2 g/dl
AMH	41		4.1–58 pmol/l (age 30–35)
Testosterone	<0.4		0.3–1.7 nmol/l
SHBG	78.1		24.6–122 nmol/l
Free testosterone index	<0.5		0.3–5.6
DHEA-S	1.8		2.7–9.2 μmol/L
25 OH Vitamin D	105		30–125 nmol/l
Vitamin B12	838		187–883 pg/ml
Folate	19.1		3–17.7 ng/ml
Progesterone		45.6	5.3–86.0 nmol/l (luteal phase)
Oestradiol		650	161–774 pmol/l (luteal phase)

Intense dysmenorrhea suggested endometriosis but financially she could not afford the cost of laparoscopy to diagnose and treat this potential disease, so surgical intervention was not pursued.

Blood test results revealed hypoandrogenemia with borderline low levels of total and free testosterone and DHEA-S significantly below the normal range. The progesterone result was 45.6 nmol/l which was ovulatory and normal by day 21 standards, but suboptimal for day 7 post-ovulation as we explain later, in the discussion.

### 2.4 Therapeutic intervention

We implemented a multifactorial treatment strategy, common to RRM fertility treatment programs.

The primary medications used on the cycle of conception included:

1. Naltrexone 4.5 mg, nightly
2. DHEA 25 mg, twice daily
3. Clomiphene 50 mg daily × 5 days from day 3 of cycle
4. HCG 10,000 iu with LH surge mid cycle
5. HCG 2,500 iu during luteal phase on days 3, 5, and 7 post-ovulation.

We initially commenced treatment with Naltrexone 3 mg nightly × 1 week to start, followed by 4.5 mg nightly thereafter to treat “clinical low endorphins” presenting with dysmenorrhea, fatigue, anxiety, and low mood. Low endorphins are associated with immune dysfunction (17) that may have contributed to previous miscarriages.

Secondly, we added DHEA 25 mg twice daily, to treat hypoandrogenemia and improve egg quality for 2 cycles pre-conception. We commonly continue DHEA 10 to 20 mg during pregnancy, titrated according to serum estradiol levels tested every 4 weeks during pregnancy.

Finally, we added medications to achieve optimal follicle function and support the luteal phase of her cycle (Table 3). She initially had follicle stimulation with Letrozole which did not achieve a balanced cycle, so we used clomiphene 50 mg daily × 5 days from day 3 of the cycle with HCG 10,000 iu when she had an LH surge followed by HCG 2,500 iu on days 3, 5, and 7 post-ovulation, based on her fertility app.

**TABLE 3:** Restorative reproductive medicine (RRM) medications and purpose.

RRM medications	Purpose
Naltrexone	To treat clinically low endorphins presenting with dysmenorrhea, fatigue, anxiety, and low mood.
DHEA 25 mg	To improve follicle function pre-conception and improve oestradiol levels during pregnancy
Clomiphene	Ovarian follicle stimulation. Titrate dose from 50 mg daily × 3 days, starting day 3 of cycle. Aim to produce one optimal follicle.
HCG 10,000 iu	Bolus HCG mid cycle timed with positive LH surge, helps follicle to rupture.
HCG 2,500 iu	Taken on days 3, 5, 7 post-ovulation to stimulate the corpus luteum and improve progesterone levels in the luteal phase of the cycle.

### 2.5 Follow up and outcomes

We used ultrasound follicle tracking scans for one cycle to confirm a single follicle developed and ruptured completely. In addition, she had monthly blood tests to confirm optimal levels of progesterone and estradiol on day 7 post-ovulation. She conceived on her second cycle of targeted intercourse during the fertile days in March 2023. Her EDD was December 2023.

We continued treatment during pregnancy with

1. DHEA 20 mg once daily
2. Cyclogest^®^ (Progesterone) pessaries 400 mg pv twice daily
3. Naltrexone 4.5 mg nightly.

She had a weekly blood test for progesterone, estradiol, and HCG for the first 3 weeks and a pregnancy scan at 7 weeks gestation to confirm viability. She continued treatment throughout the entire pregnancy as blood tests for progesterone and estradiol every 4 weeks were within normal limits with treatment. The definition of normal hormone levels and our treatment protocol were published previously (11). She delivered a healthy baby boy by 36 weeks gestation. She had an elective cesarean section, due to a thin uterine scar from the previous c-section. Birth weight was 6 lb 12 oz (3080 g). Mother and baby were both healthy.

## 3 Discussion

We identified significant hypoandrogenemia, diagnosed with low pre-ovulatory DHEA, in a 30-year-old woman with a history of five previous unexplained miscarriages, including a late loss at 24 weeks. Hypoandrogenemia is not usually considered a contributory factor for couples with recurrent miscarriage and in this case, it was identified along with clinical endorphin deficiency and poor follicle function.

Poor follicle function is assessed by evaluating ChartNeo data provided by the patient and post-ovulatory hormone levels. The ChartNeo data facilitates accurate timing of the blood test on day 7 post-ovulation. In this case, oestradiol was normal, but post-ovulatory progesterone was interpreted as sub-optimal, especially since she was receiving luteal phase HCG support. When either oestradiol or progesterone are low, it is an indication of suboptimal CL function and since the CL arises from the ovulatory follicle, we use this diagnostic evaluation to measure what we judge to be the quality of ovulation. We base this on the maximum surge of progesterone and estradiol when tested on day 7 post-ovulation. If progesterone is below 60 nmol/l, it is interpreted as ovulatory, but sub-optimal (18).

Excess or insufficient androgens are associated with poor ovarian activity and adverse reproductive outcomes (19). When hypoandrogenism exists pre-conception, androgen levels and ovarian function take time to be normalized and conversion of supplemented DHEA to testosterone can vary (20). In our experience it takes 8–10 weeks of treatment before follicle function improves, so couples are advised to avoid conceiving for the first 2 cycles. If after two cycles, optimal progesterone and estradiol on day 7 post-ovulation is achieved with DHEA treatment, the patients are encouraged to begin attempting conception. In most cases, a mild ovarian stimulation protocol is also needed to achieve optimal follicle function. This patient required clomiphene during the follicular phase, an HCG trigger shot to ensure rupture of the follicle, and luteal support with HCG. A vital part of the protocol is a reliable means of confirming ovulation (21) and precise timing of blood collection each cycle. With the ChartNeo tool we can confirm the previous low levels have been restored to normal parameters with treatment. Low circulating androgens can be easily diagnosed and treated with DHEA, making it a useful routine assessment prior to conception, for recurrent pregnancy loss.

The etiology of recurrent miscarriage is rarely a single factor, and we frequently find the cause of miscarriage to be multi-factorial. In addition to hypoandrogenemia pre-conception, our patient also had symptoms of low endorphins treated with low dose naltrexone and she required continued supplementation with DHEA during pregnancy to maintain adequate estradiol levels. Prior to conception, DHEA supplementation was shown to improve spontaneous pregnancies, a measure of improved follicle function, in women with low ovarian reserve (8). Importantly, however, we find doses of 10–50 mg/day to be effective with the lowest levels of side effects, such as acne. After conception, DHEA supplementation increases estradiol (11). Since low levels of estradiol are associated with miscarriage (22), circulating estradiol levels determine the need to continue DHEA supplementation throughout pregnancy. Since estradiol did not rise above normal, we continued DHEA supplementation (11).

Unlike thalidomide or diethylstilbestrol, DHEA is a naturally occurring substance that is only given to maintain estradiol levels during pregnancy. Progesterone is routinely supplemented in IVF and RRM patients during pregnancy but not estradiol despite serum estradiol levels being lower in patients who experience miscarriage including those who are receiving exogenous progesterone (23). Since supplementation of estradiol is associated with an increased risk of thrombosis, DHEA can serve as an effective pro-hormone for increasing estradiol production (24). After the placenta begins hormonal support, which occurs around 10 weeks of gestation, DHEA is vital for placental estrogen which becomes the primary site of production (12). But the observation that even post-menopausal women have an increase in estrogen with DHEA (24) indicates that more research is needed to determine if there are other sources of estrogen before 10 weeks of gestation besides the CL.

The utility of the NeoFertility cycle tracking app (16) was particularly valuable in this case because of the language barrier. During treatment the patient documented her fertility cycle pattern, medications, and blood results. This allowed a record of her monthly response to treatment, and she could record negative symptoms or side effects from medications as they occurred.

Since this couple desire to have more children, we will attempt to reduce excessive stress or sleep deprivation to try and improve DHEA levels through lifestyle changes. Then we will repeat the same strategy to ensure she has optimal treatment before and after conception.

After 5 pregnancy losses and extensive investigation at a recurrent miscarriage clinic without a diagnosis, we identified hypoadrogenemia as a contributing factor and successfully treated the patient with a multi-factorial protocol including DHEA before and after pregnancy resulting in live birth.

## Patient consent

We received the patient’s written consent to submit her case for publication.

## Data availability statement

The original contributions presented in the study are included in the article/Supplementary material, further inquiries can be directed to the corresponding author.

## Ethics statement

Ethical approval was not required for the studies involving humans because this is a case report of a patient receiving usual treatment in my practice. The studies were conducted in accordance with the local legislation and institutional requirements. Written informed consent for participation was not required from the participants or the participants’ legal guardians/next of kin in accordance with the national legislation and institutional requirements because the patient provided written consent to review and publish her de-identified records. Written informed consent was obtained from the individual(s) for the publication of any potentially identifiable images or data included in this article.

## Author contributions

PB: Conceptualization, Data curation, Investigation, Methodology, Writing – original draft, Writing – review and editing. CT: Formal Analysis, Methodology, Project administration, Writing – original draft, Writing – review and editing. CP: Investigation, Project administration, Resources, Writing – original draft, Writing – review and editing.
